# Progesterone administration for luteal phase deficiency in human reproduction: an old or new issue?

**DOI:** 10.1186/s13048-015-0205-8

**Published:** 2015-11-19

**Authors:** Stefano Palomba, Susanna Santagni, Giovanni Battista La Sala

**Affiliations:** Centre of Reproductive Medicine and Surgery, Arcispedale Santa Maria Nuova - IRCCS, Viale Risorgimento 80, 42123 Reggio Emilia, Italy; Centre of Reproductive Medicine and Surgery, Arcispedale Santa Maria Nuova - IRCCS, University of Modena and Reggio Emilia, Via Università 4, 41100 Viale Risorgimento 80, 42123 Modena, Italy

**Keywords:** ART, Endometrium, Implantation, Luteal phase deficiency, Progesterone

## Abstract

Luteal phase deficiency (LPD) is described as a condition of insufficient progesterone exposure to maintain a regular secretory endometrium and allow for normal embryo implantation and growth. Recently, scientific focus is turning to understand the physiology of implantation, in particular the several molecular markers of endometrial competence, through the recent transcriptomic approaches and microarray technology. In spite of the wide availability of clinical and instrumental methods for assessing endometrial competence, reproducible and reliable diagnostic tests for LPD are currently lacking, so no type-IA evidence has been proposed by the main scientific societies for assessing endometrial competence in infertile couples. Nevertheless, LPD is a very common condition that may occur during a series of clinical conditions, and during controlled ovarian stimulation (COS) and hyperstimulation (COH) programs. In many cases, the correct approach to treat LPD is the identification and correction of any underlying condition while, in case of no underlying dysfunction, the treatment becomes empiric. To date, no direct data is available regarding the efficacy of luteal phase support for improving fertility in spontaneous cycles or in non-gonadotropin induced ovulatory cycles. On the contrary, in gonadotropin in vitro fertilization (IVF) and non-IVF cycles, LPD is always present and progesterone exerts a significant positive effect on reproductive outcomes. The scientific debate still remains open regarding progesterone administration protocols, specially on routes of administration, dose and timing and the potential association with other drugs, and further research is still needed.

## Background

Multiple uses of progesterone and progestogens for women’s health in clinical practice are recognized. In obstetric care they are used for treating abortion and preterm labor prevention, and in gynecology to balance estrogens in hormonal replacement therapies or in oral contraception, and as palliative care for the treatment of gynecological malignancies. On the other hand, the role of progesterone for luteal phase support still represents a controversial topic, due to its wide and empirically transverse clinical use, from natural ovulatory cycles to assisted reproductive technologies (ARTs). Most of these controversies derive from too much confusion existing in the knowledge of corpus luteum function, progesterone production, implantation window and endometrial competence and, consequently, about luteal phase deficiency (LPD) and support.

LPD is described as a condition of insufficient progesterone exposure to maintain a normal secretory endometrium and allow for normal embryo implantation and growth [1]. This definition has been sustained during years from its first description [2].

After several years where endometrium has received much less attention in reproductive studies compared to ovary and embryo development, the endometrium is now receiving the research attention it deserves, due to its physiological importance in reproduction [3]. In fact, embryo implantation represents a critical step of the reproductive process consisting of a unique biological phenomenon [4]. Successful implantation requires a receptive endometrium, a functional embryo at the blastocyst stage and a synchronized dialog between maternal and embryonic tissues [5]. The human endometrium undergoes a complex series of organized proliferative and secretory changes in each menstrual cycle and exhibits only a short period of receptivity, known as the ‘implantation window’ [6]. Endometrial receptivity during the implantation window requires a close cooperation of an extremely large number of different factors; unfortunately, the individual role of each factor in the network of endometrial development is still not completely understood [4].

The aim of this descriptive review will be to provide clinicians with a brief document based on the best and current scientific evidences, summarizing the actual knowledge regarding the role of progesterone in implantation physiology, and to show the potential and correct use of progesterone and progestogens for treatment of LPD in the different clinical settings. To obtain evidence-based data, we performed a systematic search for studies (articles and/or abstracts), without English language limitation, collecting and analyzing the published articles in literature until July 2015. We searched on Medline (through PubMed), with the combination of the following medical subject headings or keywords: “luteal phase deficiency”, “window of implantation”, “endometrial competence”, “progesterone AND luteal phase support”, “assisted reproductive technologies AND progesterone”, “controlled ovarian hyperstimulation AND luteal phase support” and “frozen-thawed cycles AND luteal phase support”. Additional literature searches were performed on the references from the identified studies. We gave priority to meta-analysis, systematic reviews and randomized clinical trials (RCTs), based on the personal evaluation of each author. When meta-analytic data or data from RCTs were lacking, prospective non-randomized and then cohort studies were included in the final analysis.

## Implantation physiology

The uterus differentiates into an altered state when implantation-competent blastocysts are ready to initiate implantation. This state is called uterine receptivity for implantation, i.e. the implantation window, and lasts for a limited time. At this stage, the uterine environment is conducive to blastocyst growth, attachment and the subsequent events of implantation. The main hormones involved in uterine receptivity are the ovarian steroids, progesterone and estrogen. Uterine sensitivity to implantation is classified into pre-receptive, receptive and non-receptive (refractory) phases. In humans, the endometrium is classified histologically and functionally into proliferative and secretory phases during the average 28–30 day menstrual cycle. During the secretory phase, the uterus is considered pre-receptive for the first ~7 days following ovulation; it then becomes receptive during the mid-secretory phase, which spans 7–10 days after ovulation; the non-receptive (refractory) phase comprises the rest of the secretory phase [7].

Molecular and genetic evidence indicates that locally produced signaling molecules, including cytokines, growth factors, homeobox transcription factors, lipid mediators and morphogens, together with ovarian hormones serve as autocrine, paracrine and juxtacrine factors to obtain uterine receptivity [8]. Therefore, the implantation mechanism includes the cooperation of several factors, such as the integrins on both blastocyst and receptive endometrium surfaces, progesterone which stimulates the endometrium receptivity and the decidulization of stroma and a series of cytokines, such as IL-1 and EGF, which participate in the regulation of endometrial immune tolerance, expression of integrin nd prostaglandins production [7, 8].

The corpus luteum is derived from the transformation of granulosa and theca cells into luteal cells in response to the mid-cycle surge of gonadotropins or to an exogenous human chorionic gonadotropin (hCG) bolus administration. The most important function of the corpus luteum is progesterone secretion which is necessary to obtain a secretory transformation of the endometrium and to sustain the early pregnancy. Progesterone prepares the endometrium for pregnancy by stimulating proliferation in response to hCG. This occurs in the luteal phase of the menstrual cycle.

During the midluteal phase of a natural cycle, progesterone is involved in the modulation process of the expression of ultrastructural hallmarks of secretory transformation, such as giant mitochondria, subnuclear glycogen deposits, pinopodes and nucleolar channel system (NCS) [9]. With the aim of inducing endometrial competence/receptivity, progesterone can also act by stimulating the immune system to produce non-inflammatory T-helper 2 cytokines and C3-C4, as shown in patients with autoimmune diseases [10, 11], increasing nitric oxide production, with improvement of the blood flow and oxygen to the endometrium [12]. Progesterone is also able to reduce the contractility of the myometrium at the time of the implantation [13].

The crucial role of the corpus luteum in human reproduction in the maintenance of early pregnancy is demonstrated by the harmful effects of a lutectomy during the first weeks of a pregnancy. Initial studies on corpus luteum function demonstrated that, in patients at 7 weeks of pregnancy, after a tubal ligation, the plasma progesterone levels were normal and pregnancy continued but, after a tubal ligation plus lutectomy, the plasma progesterone levels dropped to near zero within 5 days and the pregnancy was aborted [14]. In this week of gestation, after tubal ligation and lutectomy, progesterone replacement was effective in maintaining the pregnancy. At and after 8 weeks of amenorrhea, tubal ligation plus lutectomy decreased plasma progesterone levels until day 4 but these then increased to pre-treatment concentrations with successful continuation of pregnancy [14].

This “genuine” endometrial competence, however, can be negatively affected in clinical practice by several functional diseases, such as polycystic ovarian syndrome (PCOS) and obesity [15] and anatomic abnormalities, such as congenital uterine anomalies [16], endometrial polyps [17], myomas (submucosal) [18], hydrosalpinx [19] and endometriosis [20]. In fact, the treatments of these diseases have been discussed as potential therapies to improve fertility and clinical pregnancy rate in spontaneous and/or in in-vitro fertilization (IVF) cycles. The pregnancy rate was found to be two-fold higher [relative risk (RR) 2.0, 95 % confidence interval (CI) 1.1 to 3.9] after myomectomy of submucosal myomas [18] or after polipectomy of 3–24 mm endometrial polyps (RR 2.1, 95 % CI 1.5 to 2.9) [21]. Laparoscopic salpingectomy for hydrosalpinges versus no-surgical treatments improved the clinical pregnancy rate by more than two-fold [odds ratio (OR) 2.49, 95 % CI 1.60 to 3.86) [19]. Finally, compared with diagnostic laparoscopy, laparoscopic surgery for endometriosis was associated with a significant improvement of the clinical pregnancy rates (OR 1.89, 95 % CI 1.25 to 2.86) [20].

## Endometrial competence evaluation

Endometrial competence can be assessed using different methods, such as histology, timing, ultrasound and biomarkers.

In consideration of the LPD definition, histological evaluation of the endometrium has been considered the gold standard procedure to study endometrial competence. The histological method is based on a careful examination of an endometrial biopsy, and on the definition of the histologic characteristics of a secretory endometrium and describing the temporal responses to progesterone, i.e. endometrial dating. An endometrium is considered out of phase when there is a lag of more than 2 days [22]. Over the years, the histological method has developed more specific instruments, such as scanning electron microscopy [23, 24] and the immunohistochemical analysis of estrogen and progesterone receptors [25]. However, randomized clinical trials (RCTs) suggest that endometrial biopsy is an inaccurate tool for differentiating fertile women from women with LPD and infertility [1].

The timing as criteria of endometrial competence is based on LH peak, ultrasound demonstration of ovulation, basal body temperature shift and menstrual flow after the biopsy but it also presents a wide inter- and intra-subject variability [1, 26].

Ultrasound assessment is still used to evaluate endometrial competence though no significant correlation has been seen between endometrial measurement (>7 mm) and the pregnancy rate; moreover, the triple-layered pattern is visible in the same rate in infertile and fertile women (91 % vs. 90 %, respectively) [27]. In order to increase accuracy of the ultrasound method and to find a predictor sign of pregnancy during IVF-treatment, the power-Doppler was proposed as the instrument for evaluating endometrial competence, but no statistically significant differences in the vascularization indexes of endometrial and sub-endometrial blood flows were found between pregnant and non-pregnant groups [28].

Several molecular markers of endometrial competence have been discovered in blood and uterine fluid analysis and endometrial biopsy, such as estradiol, progesterone, pinopodes, glycodelin, IL-1 system, cytokines, integrins and HOXA genes [29]. Serum progesterone assay has been proposed as surrogate of endometrial competence but it has been observed that no minimum progesterone levels can define a “fertile” luteal phase [30] because progesterone concentrations may fluctuate up to eightfold within 90 min [31] and its levels peak 6 to 8 days after the ovulation [32].

Among the over one hundred different genes identified, all potential biomarkers of the implantation window, few studies exist with adequate power and validity based on study design to help determine which biomarkers have the greatest value and consistency; therefore, no reliable methods to assess “receptivity” have been established or adequately validated [33]. Nevertheless, the transcriptomic approaches and microarray technology make it possible to identify biomarkers of endometrial receptivity and to report modifications related to the gene expression profile associated with the transition of the human endometrium from a pre-receptive to a receptive state during a natural cycle and, most of all, to reveal either moderate or strong alterations of endometrial receptivity under controlled ovarian stimulation (COS) protocols [34]. In fact, a study analyzed the endometrial gene expression profiles by paired samples from the same patients during the pre-receptive to the receptive transition both in a natural and a subsequent stimulated cycle [34]. This study has shown that COS regimens affect the transcriptomic pattern of endometrial cells in comparison with natural cycles; in particular, there were numerous differences in the main systems involved in the implantation process, such as the TGFb signaling pathway, the complement and coagulation cascades and leukocyte transendothelial migration [34]. This information could open new perspectives, particularly in patients with multiple implantation failures [34]. A recent study revealed that some cases of repeated implantation failure could be associated with an aberrant gene expression profile, particularly of transcripts related to the immune function and complement activation, and altered progesterone signaling might be an underlying mechanism for such endometrial gene expression deregulation in women with repeated implantation failure [35]. Other research has been focused on the family of fibroblast growth factors (FGFs), which affects embryo implantation and supports improved endometrial trophoblastic interaction [36]. Compared with the fertile group, FGF-1 is not expressed strongly enough in the failed IVF patients, which may have caused a lack of endothelial cell migration, important for implantation, stopped the process of blood vessel formation or induced early vascularization of implantation problems [37].

However, in the current state, no type-IA evidence has been proposed by the main scientific societies for assessing endometrial competence in infertile couples. In fact, the Royal College of Obstetricians & Gynecologists (RCOG) guidelines for infertility do not recommend the use of basal body temperature charts nor an endometrial biopsy to evaluate the luteal phase in routine infertility investigation [38]. The blood test measurement of serum progesterone in the mid-luteal phase of the cycle is recommended but with type-B evidence [38]. At the same time, according to the recommendation of the American Society for Reproductive Medicine (ASRM), no diagnostic test for luteal phase insufficiency has been proven to be reliable in a clinical setting [1].

## Luteal phase support in clinical practice

The discussion regarding the clinical role of LPD is mainly due to the lack of reproducible and reliable diagnostic tests. Nevertheless, LPD is a common condition, that occurs during a series of clinical patterns characterized by low follicular-stimulating hormone (FSH) levels, altered follicular FSH/luteinizing hormone (LH) ratio and/or abnormal FSH and LH pulsatility, such as functional hypothalamic amenorrhea, thyroid and prolactin disorders [39], obesity and PCOS [15] and during COS for IVF cycles [40]. Therefore, the correct approach to the treatment of LPD is the identification and correction of any underlying condition. On the other hand, in case of no underlying dysfunction, the treatment becomes empiric [1].

Notwithstanding the empiricism of the LPD treatment, there are different clinical conditions where luteal phase support may be more or less useful, and can drive the choice for a specific luteal phase support.

## Spontaneous ovulatory cycles

In spontaneous cycles there are no direct data on the efficacy of luteal phase support for improving fertility. A recent Cochrane meta-analysis on miscarriage prevention showed no statistically significant difference between women receiving progestogens (either natural and synthetic) and those receiving only placebo or no treatment (OR 0.99, 95 % CI 0.78 to 1.24) [41]. The same meta-analysis found a statistically significant reduction (OR 0.39, 95 % CI 0.21 to 0.72) in miscarriage rate after progestogen administration only in women with recurrent miscarriage, i.e. three or more consecutive miscarriages. Moreover, these findings deserve further studies because the trials included were of poorer methodological quality [41]. Also for the treatment of threatened miscarriage, there is insufficient evidence to support the routine use of progestogens (whether natural or synthetic) [42]. In conclusion, in natural and unstimulated cycles, no treatment for LPD has been shown to improve pregnancy outcomes [1].

## Non-gonadotropin induced ovulatory cycles

The non-gonadotropin induced ovulatory cycles mainly use clomiphene citrate (CC), while aromatase inhibitors (AIs) and metformin are still considered experimental treatments and to be employed in selected cases, respectively.

CC is a nonsteroidal selective estrogen modulator with both estrogenic and anti-estrogenic properties that interferes with the normal feedback mechanisms and leads to increased and prolonged FSH and LH secretion, which in turn stimulates follicular development. Cumulative CC administrations induce weak and prevalent anti-estrogenic effects on sensitive tissues, such as endometrium and/or ovary-related luteal phase defect and/or folliculogenesis alterations [43]. The competitive binding of the CC with estrogen receptors makes the estrogenic sensitive organs less-sensitive to endogenous and exogenous steroid [43]. Moreover, CC administration appears to be related to a reduced perifollicular vascularization and to a rate of high grade follicles significantly lower than in healthy controls [44]. Also the vascularization of corpus luteum is significantly lower and with higher resistance in CC cycles, considering this hypo-vascularization of corpus luteum as a possible LPD cause [44].

To this regard, several data are available in literature about luteal phase progesterone support after ovulation induction in intrauterine insemination (IUI) cycles. A recent meta-analysis concluded that progesterone support did not benefit the clinical pregnancy rate in patients undergoing ovulation induction with CC (RR 0.89, 95 % CI 0.47 to 1.67) nor a significant difference in miscarriage per cycle between the two groups (OR 1.03, 95 % CI 0.52 to 2.04) [13]. Of note, no heterogeneity in the findings obtained was detected and there were no data on live birth to perform data synthesis. The same results were obtained in another recent meta-analysis [45]. In CC-stimulated cycles, progesterone administration did not improve the reproductive outcomes, such as the live birth rate (RR 1.30, 95 % CI 0.68 to 2.50) and the clinical pregnancy rate (RR 1.17, 95 % CI 0.82 to 1.65) and no differences were observed regarding miscarriage rate between the progesterone-treated and not-treated groups in the CC stimulation protocol (RR 1.14, 95% CI 0.63 to 2.06) [45].

In CC-stimulated cycles, the combined administration of estrogens and progesterone was tested in clinical studies showing not univocal results. In fact, endometrial estrogen receptors are blocked by competitive binding of the CC. Thus, they could be not stimulating by estrogens. Moreover, a RCT demonstrated that ethinylestradiol administration from day 8 for 5 days (0.05 mg daily) significantly improves the efficacy of progesterone support administered intramuscularly [46]. Similarly, another RCT showed a significant reproductive benefit of oral estradiol administration (3 mg daily in two administrations from cycle day 8 until ovulation) followed by vaginal progesterone in CC cycles [47]. Finally, a very recent randomized, double-blind, placebo-controlled trial compared in PCOS women the clinical pregnancy rate between two groups treated with CC plus ethinyl estradiol and CC plus placebo, respectively. The study resulted in an increase of clinical pregnancy rate in the CC + EE group (29 % vs 10 %, p = 0.02) even if there was no statistically significant difference in the ongoing pregnancy rate between the two groups [48]. However, an interesting recent retrospective cohort analysis revealed that luteal phase progesterone supplementation in CC-IUI cycles can improve endometrial receptivity with an effect closely related to the endometrial thickness [49]. Patients who appeared to receive the greatest benefit of progesterone supplementation had an endometrial thickness of 6–8 mm; their clinical pregnancy rate was found to be improved two-fold (OR 2.04; 95 % CI 1.01 to 4.14) [49]. These patients seem to have an endometrium still receptive to progesterone administration, whereas patients with an endometrial thickness less than 6 mm are not responsive to progesterone supplementation for CC-related receptors depletion/inhibition and patients with an endometrial thickness greater than 8 mm represent a group with good reproductive prognosis in which progesterone supplementation is unable to provide further reproductive improvement.

Metformin improves the regular menstrual cycles and increases ovulation rate in patients with PCOS, although the efficacy of the drug is extremely variable both between different PCOS populations and within the same population [50]. The efficacy of metformin in inducing ovulation in patients with PCOS is probably due to a direct action of the drug on the ovary; in fact, the ovulatory response to the drug seems to be related more to local drug sensitivity or resistance than to improvements in the systemic hormonal and/or metabolic environments [51], as shown by the analysis of follicular fluid that seemed to confirm that metformin acts directly on the ovary, improving local levels of androgens, ovarian insulin resistance and the levels of several growth factors [50]. As regards the use of metformin as ovulation inductor, unfortunately few and no direct data exist concerning the need of a luteal phase support. Under metformin treatment, the hormonal pattern and the ovarian dynamics of the ovulatory cycles were found to be similar to spontaneous cycles as observed in normo-ovulating controls [52]. In addition, the preovulatory follicles obtained under metformin treatment had a vascularization similar to that observed during natural cycles of healthy women, and the rate of high grade preovulatory follicles observed in women with PCOS who ovulated with metformin was not significantly different from controls [44]. Finally the vascularization around the corpus luteum was found to be similar between metformin and spontaneous cycles, confirming the beneficial effects of metformin on the corpus luteum function [44].

Letrozole is an aromatase inhibitor recently employed as ovulation inducer. A recent large multicenter randomized double-blind parallel controlled trial, published in 2014, demonstrated the superiority of letrozole as first-line therapy for anovulatory infertility in women with PCOS when compared with CC [53]. A systematic review of RCTs with meta-analysis concluded that letrozole is associated with significantly higher live birth rates than with CC (OR 1.64, 95 % CI 1.32 to 2.04) and with significantly higher clinical pregnancy rate compared to CC (OR 1.40, 95 % CI 1.18 to 1.65) even if the quality of the evidence was rated as low [54]. Letrozole acts by inhibiting the aromatase, a cytochrome P450 enzyme complex which is responsible for androgen to estrogen conversion, so it induces a hypoestrogenic state that stimulates, through activation of hypothalamic-pituitary axis, increased FSH secretion and ovarian follicle development [43]. It does not exert an antagonist effect on endometrial estrogen receptors; on the other hand suppression of the systemic estrogen levels and in peripheral tissues can result in up-regulation of the estrogen receptors in the endometrium, leading to rapid endometrial growth once follicle development starts and estrogen secretion is restored [43].

## Controlled ovarian stimulation (COS) with gonadotropins for non-IVF cycles

In non-IVF cycles with gonadotropins COS, two recent meta-analyses [45, 46] have demonstrated the benefit in improving reproductive outcomes with vaginal progesterone use as luteal phase support. Specifically, in the first meta-analysis, the clinical pregnancy rate was increased to 70 % (OR 1.77, 95 % CI 1.20 to 2.60), as well as the likelihood of live birth per cycle (OR 2.63, 95 % CI 1.42 to 4.80), in patients receiving progesterone support [45]. In the second meta-analysis, patients treated with FSH showed higher biochemical pregnancy (RR 1.81, 95 % CI 1.36 to 2.43), clinical pregnancy (RR 1.57, 95 % CI 1.15 to 2.15) and live birth (RR 2.28, 95 % CI 1.49 to 3.51) rates on receiving progesterone supplementation [46]. No differences were observed regarding miscarriage rate between the two study groups (progesterone treated and not-treated groups) [46]. In both meta-analyses [45, 46], data heterogeneity was low and not significant.

To the regard of the optimal progesterone dosage to use in non-IVF COS cycles with gonadotropins, very few clinical evidences are available in literature. A very recent RCT, evaluating two doses of vaginal progesterone for IUI cycles in terms of pregnancy rates, demonstrated that 300 mg of intravaginal micronized progesterone should be the maximum dosage for luteal phase when compared with 600 mg [55].

## Controlled ovarian hyperstimulation (COH) with gonadotropins for IVF cycles

To explain in detail the role of progesterone and/or progestogens for the luteal phase support in gonadotropins COH for IVF cycles, the issue should be approached considering conventional and new protocols of gonadotropins COH separately.

### Conventional COH protocols

Conventional COH protocols with gonadotropins have seen the transition over time from use of depot GnRH-agonist plus high-dose gonadotropins (225 IU/daily) in step-down or chronic regimen to daily GnRH-agonist *plus* low-doses gonadotropin (150 IU/day) in the patients’ response adjusted regimen, i.e. a “mild stimulation”. Ovarian hyper-stimulation was seen to affect embryo quality as assessed by morphology as well as the chromosomal constitution of the embryos [56–58], as result of interference with the natural selection of good-quality oocytes or the exposure of growing follicles to the potentially negative effects of ovarian stimulation [59]. Mild stimulation approaches, aiming at a more physiological response, seem to improve embryo quality [59]. In fact, a meta-analysis combining the results of three separate RCTs suggests that the retrieval of a reduced number of oocytes following mild stimulation is associated with a higher implantation rate compared with patients where the same number of oocytes is retrieved following conventional stimulation [60]. Mild stimulation approaches might improve also embryo implantation rates [61].

In both these conventional COH for IVF cycles, an LFD is always present and it is due to supra-physiological estradiol levels which inhibit LH release, as negative feed-back mechanism, and alter endometrial responsiveness to progesterone [62]. Moreover, the use of GnRH agonist [63] and antagonist [64] implies luteolysis and decreased LH pulsatility because of a competitive receptor’s blockage.

As result, in conventional IVF cycles, after hCG trigger, hCG levels rapidly increase until maximum levels at the time of oocyte retrieval, before falling to the lowest levels approximately 5 days after the retrieval [65]. The trend of progesterone follows that of hCG more slowly, with a peak near to 5 days after oocyte retrieval, then decreases rapidly [66]. Therefore, it creates a window during which progesterone lacks hCG stimulation to reach the threshold of 80–100 nmol/L, necessary to maintain the pregnancy [67]. The luteal support is required in this time interval.

The main guidelines of the scientific society agree to recommend exogenous progesterone supplementation in assisted reproductive technologies (ARTs) cycles [68, 69]. A recent Cochrane meta-analysis regarding luteal phase support for ARTs cycles confirmed that progesterone exerts a significant positive effect on clinical pregnancy (OR 1.89, 95 % CI 1.30 to 2.75) and on pooled live birth or ongoing pregnancy (OR 1.77, 95 % CI 1.09 to 2.86) rate [70]. However, when the analysis was restricted to live birth rate alone, evidence suggested no significant effect of the progesterone administration (OR 4.21, 95 % CI 0.93 to 19.18) [70]. Surprisingly, in a previous Cochrane review [71] the same authors concluded that progesterone had a significant positive effect on live birth rate compared to placebo, although in both reviews the effect of progesterone on the live birth derived from the results of a single study. In the most recent Cochrane [70], no significant data heterogeneity was detected, although the quality of the evidence was considered as very low to low. On the other hand, even if the hCG administration for luteal phase support is also effective, i.e. no differences between progesterone and hCG in rates of live birth or ongoing pregnancy (OR 0.95, 95 % CI 0.65 to 1.38), its use increases the ovarian hyperstimulation syndrome (OHSS) risk by more than four-fold compared to placebo (OR 4.28, 95 % CI 1.91 to 9.60) and compared to the progesterone group (OR for OHSS of progesterone group vs. hCG group: 0.50, 95 % CI 0.34 to 0.76) [70].

Progesterone has different pharmacokinetic and pharmacodynamics properties when used in different routes of administration. In fact, intramuscular progesterone in oil formulation is related to higher serum progesterone concentrations and lower endometrial concentrations (unlike vaginal route) [72]. In addition, in consideration of a half-life longer than one day, a depot effect with a continuous release over time without intermittent peaks (as in the case of vaginal route) has been observed for intramuscular progesterone [73]. These pharmacokinetic properties of intramuscular route allow a wider implantation window and less endometrial contractions per minutes on the day of the embryo transfer (1 vs. more than 4 endometrial waves) [73]. Unfortunately, intramuscular progesterone in oil formulation is available only in a few countries and represents a very painful route of administration. Similarly, the other available options for delivering exogenous progesterone have other restrictions, such as a low absorption rate for oral administration, due to the first liver-pass effect and potential side effects due to its metabolites, or the need for daily repeated administrations of vaginal progesterone, which can result in an uncomfortable route and with absorption variability due to leakage [74]. Different routes of progesterone administration have been compared in clinical studies. An initial meta-analysis [75] found no significant differences in treatment outcomes between vaginal and intramuscular progesterone administration. More recently, also a new systematic review and meta-analysis [70] showed that there were no statistically significant differences in live birth rate between intramuscular vs. oral (OR 0.71, 95 % CI 0.14 to 3.66) and vs. vaginal or rectal (OR 1.31, 95 % CI 0.84 to 2.05) route. The same statistical inconsistence remained between the different routes of administration also when other intermediate endpoints, such as the clinical pregnancy rate, were evaluated [70]. Moreover, results were obtained after data extrapolation from few studies on little study’ populations [70].

Regarding to the dose of progesterone, the most recent Cochrane review demonstrated no differences in live birth or ongoing pregnancy rate (OR 0.97, 95 % CI 0.84 to 1.11) between standard (90 mg/day) or high (equal or more than 100 mg/day) doses of vaginal progesterone without heterogeneity among studies [70]. Moreover, the RCTs synthesized included different formulations and were of suboptimal quality. Both histopathological [76] and clinical studies [77] suggested that vaginal progesterone in doses lower than 300 mg/day are less efficacy to intramuscular progesterone (100 mg daily) whereas no difference in efficacy was demonstrated when doses of 600 mg/day of vaginal progesterone were used [78]. In addition, another recent RCT comparing low-dose of micronized vaginal progesterone (200 mg twice daily) versus high-dose (200 mg three times daily) confirmed that the first regimen is less effective in term of clinical and ongoing pregnancy [79]. Moreover, a noninferiority RCT found no statistical differences in clinical pregnancy rate, implantation rate and miscarriage rate between intramuscular (50 mg/day) and vaginal progesterone, when administrated as micronized progesterone bioadhesive gel both at standard (90 mg/day) and high-dosage (90 mg twice a day) [80]. The efficacy of micronized progesterone in bioadhesive gel formulation (90 mg/day) was also confirmed in a multicentre RCTs demonstrating no statistical differences in live-birth rate and pregnancy rates when compared with other vaginal formulations (100 mg), although used at higher dosages [81, 82].

The goals for new progesterone treatment seem to be represented by aqueous subcutaneous progesterone. Two large multicentre RCTs demonstrated that the 25 mg subcutaneous progesterone administered once daily is effective and well tolerated [83, 84]. Both 25 and 50 mg daily doses of aqueous subcutaneous progesterone led to similar secretory transformation of endometrium in reproductive-aged women who were down-regulated with GnRH agonist and treated with estradiol [85] suggesting that 25 mg should be considered the lowest effective dose. The subcutaneous administration maximizes the kinetic profile and the physiological response, i.e. lower dose needed, minimizing side effects [85, 86]. In fact, it reduces patient discomfort (fewer skin reactions) and it may be appealing to women who prefer to avoid the intramuscular and vaginal routes of administration [84]. This dose also relates to the daily production of progesterone, which has been estimated to be 25 mg.

Other compounds were assessed alone or in combination with progesterone for luteal phase support in conventional IVF cycles. As explained earlier, hCG should be avoided as it leads to a higher risk of OHSS (see above) and the addition of estrogens to progesterone did not show a better effect than progesterone alone in the rates of ongoing pregnancies (OR 1.12, 95 % CI 0.91 to 1.38) and live births (OR 1.32, 95 % CI 0.93 to 1.86) [70]. The only significant result was found in the comparison of progesterone versus progesterone plus GnRH agonists [70]. Specifically, the addition of GnRH agonist to progesterone had a significant greater effect than progesterone alone in live birth rate (OR 0.40, 95 % CI 0.26 to 0.61), clinical pregnancy rate (OR 0.71, 95 % CI 0.59 to 0.87) and pooled live birth/ongoing pregnancy rate (OR 0.67, 95 % CI 0.56 to 0.81) [70]. Although a high statistical heterogeneity was detected in this analysis, the direction of the effect was considered consistent. The effectiveness of the GnRH agonist administration in luteal phase support has been confirmed both in GnRH agonist and GnRH antagonist COHs [87]. Moreover, the luteal support with GnRH agonist requires repeated daily administrations. In fact, due to the pharmacokinetics of the GnRH agonist-induced LH releases (see below), a clear-cut positive correlation between the frequency of GnRH agonist administration (i.e., buserelin) and serum progesterone levels was observed [88, 89].

In literature, there is a debate regarding also the timing of progesterone administration, once a decreased likelihood of pregnancy has been observed if progesterone is initiated both before oocyte retrieval or 6 days after oocyte aspiration [90]. A systematic review of 5 RCTs with a total of 872 patients and 1010 cycles shows the presence of a window for progesterone start, occurring between the evening of oocyte retrieval and day 3 after oocyte retrieval, when the embryo-to-endometrial synchrony and exogenous luteal phase support seem to be optimized [90]. Of these RCTs, only one reported live birth rates, finding no differences in live birth between patients randomized to receive progesterone 36 h before oocyte retrieval, the evening of the oocyte aspiration or day 3 after oocyte retrieval [91] even if this study was not powered to detect a difference in live birth rate. In fact, all studies reported the clinical pregnancy rate as a primary outcome. A lower clinical pregnancy rate was detected in patients starting progesterone 12 h before oocyte retrieval compared with those patients starting progesterone the evening of oocyte retrieval (12.9 % vs. 24.6 %, respectively; *P* = 0.01) [92]. Similarly, a lower clinical pregnancy rate was observed in patients undergoing fresh autologous IVF and starting progesterone 6 days after oocyte retrieval when compared with starting progesterone 3 days after oocyte retrieval (44.8 % vs. 61.0 %, respectively; *P* = 0.05) [93]. Finally, the last three studies that compared the clinical pregnancy rate in patients starting progesterone the evening of oocyte retrieval versus 2 days after and 3 days after oocyte retrieval did not detect significant differences between the groups [91, 94, 95].

The optimal duration of progesterone supplementation after IVF cycles has also been the object of debate. A meta-analysis assessed the effects of different lengths of progesterone treatment [96]. No statistical difference in live birth (RR 0.95, 95 % CI 0.86 to 1.05), miscarriage (RR 1.01, 95 % CI 0.74 to 1.38) and ongoing pregnancy (RR 0.97, 95 % CI 0.90 to 1.05) rate between an early progesterone cessation at the first positive pregnancy test and a progesterone continuation until 6th-7th weeks of pregnancy was observed [96]. No or low heterogeneity was observed among studies in the main outcome measures [96].

### Non-conventional COH protocols

In order to optimize the safety of the COH in selected populations, such as cancers patients and donors, new COH protocols have been initially proposed, and further extended to hyper- and normo-responders to achieve the “OHSS-free clinic” dream [97]. The “non-conventional” COH protocols consist of using GnRH antagonist for avoiding LH surge and standard/high gonadotropin dosage [98, 99]. In case of no or low OHSS risk, multiple ovulation triggering can be induced by conventional hCG administration [98–103]. On the other hand, in case of moderate and/or high OHSS risk, GnRH agonist superovulation triggering followed by elective [100–103] or non-elective cryopreservation programs (segmentation of IVF cycles) [98, 99] can be an option. The “IVF cycle segmentation” consists in the cryopreservation of all embryos produced and their replacement in a receptive non-stimulated endometrium, such as in a natural cycle, or after artificial endometrial preparation [104]. This concept is also supported by positive reproductive outcomes of existing RCTs in favor of a strategy of frozen embryo transfer, although same aspects remain unclear as well as the higher incidence of “large baby syndrome” [105]. Thus, larger trials are needed before a critical change in clinical practice can be widely accepted [106].

From the first study analyzing the potential role of GnRH agonist to induce final follicular maturation, the concern of a deep LPD in these IVF cycles and the need of an intensive luteal phase support emerged [107]. The use of standard treatment to support the luteal phase after GnRH agonist triggering is considered inadequate in consideration of the lower conception rates observed [108]. In fact, GnRH agonist triggering shows a combined negative effect on the function of the corpus luteum and on the function of the endometrium [109, 110].

Relevant differences exist regarding the duration and profile of the GnRH agonist-induced surge of gonadotropins when compared with that of the natural cycle [111, 112]. The GnRH agonist-induced LH surge consists of two phases: a short ascending limb of about 4 h and long descending limb of about 20 h for a total length of 24–36 h. On the other hand, the mid-cycle LH-surge of a natural cycle is characterized by three phases: a rapidly ascending phase lasting 14 h, a plateau of 14 h and a descending phase of 20 h, for a total length of 48 h [113]. Therefore, the total amount of LH released during the surge is significantly reduced when GnRH agonist is used to trigger ovulation compared with that in natural cycle or with hCG triggering [113]. Moreover, the LPD after GnRH agonist triggering is not due to low serum LH levels but to the rapid half-life of LH. In fact, LH levels of 4–10 IU/L are sufficient to induce a physiologic peak of progesterone (25 nmol/L) [67], and even if the triggering with hCG induces a release in progesterone higher than 100–250 nmol/L, the serum progesterone levels of 80–100 nmol/L, as obtained after GnRH agonist triggering, should be sufficient to support the luteal phase [67, 114]. On the other hand, the half-life of LH is approximately 21 min vs. the half-life of hCG that is 12 h [115]. Thus, the mean duration of a non-supplemented luteal phase after GnRH trigger may be as short as 4 days, compared with 13 days after hCG trigger [116].

Finally, biological changes in endometrium related to GnRH agonist use were also observed. In particular, differences were seen in endometrial gene expression, related to the type of ovulation trigger and luteal support. The mode of triggering is reflected in the transcriptome of the somatic cells of the follicle since they differentially expressed genes like ANGPT1 and SEMA3A in mural granulosa cells. This suggests an impaired induction of angiogenesis in the GnRHa-triggered as compared with the hCG-triggered, which in association with the lower postovulatory LH activity, and may explain the insufficient luteal phase observed after COH and GnRHa trigger [117]. A RCT showed that the gene expression after GnRH-a trigger and the modified luteal support adding LH/hCG activity more closely resembles the pattern seen with the use of hCG for trigger and of a standard luteal support with vaginal progesterone [118].

The final result is a deeper LPD in GnRH agonist triggering cycles compared with hCG triggering cycles. Actually, the scientific debate still remains open regarding the role of GnRH agonist in trigger ovulation because the most recent and updated Cochrane review [119] about GnRH agonist vs. hCG for oocyte triggering in antagonist ARTs largely confirmed the same conclusions as the previous one [120]. In particular, the Authors concluded that GnRH agonist, as a final oocyte maturation trigger in fresh autologous cycles, is associated with a lower live birth rate, a lower ongoing pregnancy rate (beyond 12 weeks of amenorrhea) and a higher rate of early miscarriage (less than 12 weeks) [119]. Thus, GnRH agonist triggering could be useful and employed only for women who choose to avoid fresh transfers, women who donate oocytes to recipients or women who wish to freeze their eggs for later use [119]. In reality, a data synthesis of reproductive results may be not feasible since the studies included were not comparable due to difference in luteal phase support protocols [121].

New and different regimens have been proposed for a greater luteal phase support in GnRH-a triggering: the intensive or “American” approach which consists of an aggressive steroidal support (intramuscular or vaginal progesterone plus transdermal estradiol) with adjuvant low-dose hCG trigger only in selected cases, such as women with peak serum E_2_ less than 4000 pg/ml on the day of trigger, and the moderate or the “European” approach which promotes the production of endogenous steroids by the corpus luteum via exogenous hCG supplementation, immediately after the oocyte retrieval, at dose low enough to avoid the development of OHSS [108]. Youssef et al. [119] highlighted that the modified luteal phase support with LH/hCG (the European concept) is associated with pregnancy rates almost comparable with those of hCG triggering cycles, albeit still significantly lower. Another regimen suggested for a more sustained luteal support is the use of one bolus of 1500 IU hCG concomitant with GnRH-a (dual trigger) 34–36 h before oocyte retrieval [122, 123]. With the dual trigger, acceptable rates of implantation, clinical pregnancy, ongoing pregnancy rates, and early pregnancy loss has been achieved in high responders [122, 123], even if the incidence of clinically significant OHSS was not eliminated, but rather reduced to 0.5 % [123]. In fact, the minimal hCG activity needed for luteal phase support without inducing late-onset OHSS is not known. In a RCT, two cases of moderate OHSS out of 125 patients (considered normal responders) treated with GnRH agonist triggering plus 1500 IU hCG on the day of oocyte retrieval and an additional bolus of 1500 IU of hCG 5 days after the oocyte retrieval were reported [124]. Thus, that protocol for luteal phase should be avoided because of a persistent OHSS risk [124]. Finally, a case of recurrent empty follicle syndrome has recently been described, successfully treated by ovulation trigger with GnRH-a 40 h and hCG added 34 h prior to oocyte retrieval (double triggering) [125]. This new regimen is based on the concept of prolonging the time interval between ovulation triggering with GnRH-a and oocyte retrieval [126] with the consequent simultaneous induction of an FSH surge; thus the "double trigger" could overcome any existing impairments in granulosa cell function, oocyte meiotic maturation or cumulus expansion, resulting in successful aspiration of mature oocytes, pregnancy and delivery [127]. In line with these results, the double triggering was later offered also to two groups of patients demonstrating abnormal final follicular maturation despite normal response to COH, those with low (<50 %) number of oocytes retrieved per number of dominant follicles (i.e. > 14 mm in diameter) on the day of hCG administration [128] and those with low proportion of mature/metaphase-II (MII) oocytes (<66 %) per number of oocytes retrieved [129]. In both groups, with double triggering, patients showed significantly higher number of oocytes retrieved, higher number of mature oocytes per number of oocytes retrieved, with a tendency toward a higher number of top-quality embryos, as compared to the hCG-only trigger cycles [128, 129].

Summarizing, available evidence supports the use of GnRH agonist trigger as a helpful approach in women with moderate and/or high risk for OHSS. In fact, in women with an extreme response to stimulation a GnRH agonist trigger will be followed by a "freeze-all" strategy to eliminate OHSS risk, while in patients with a low-moderate OHSS risk the GnRH-agonist trigger followed by intensive luteal phase support could allow fresh transfer with good, albeit inferior, reproductive outcome and a significantly reduced risk of OHSS. Finally, in low OHSS risk patients, a GnRH agonist trigger with modified luteal support (hCG rescue bolus after oocyte retrieval) could be an alternative to conventional hCG trigger, given the excellent pregnancy rates [130] while GnRH-a and hCG may be offered concomitantly, 35–37 h prior to oocyte retrieval (i.e. dual trigger) or 40 h and 34 h prior to oocyte retrieval respectively, i.e. double trigger, in women with abnormal final follicular maturation [127].

Among these women, GnRH agonist trigger also introduces the possibility of an “exogenous progesterone-free” luteal phase, that relies solely on endogenous progesterone production from the corpus luteum driven by small boluses of LH activity (hCG) administered during luteal phases [130, 131]. This innovative option for luteal phase support needs to be corroborated in future large trials but it could bring an end to inconvenient vaginal discharge and/or to painful intramuscular injections of progesterone [108].

## Donor and/or frozen-thawed cycles

Due to increased mean age of maternity in developed countries, IVF using donor oocytes is an increasingly used infertility treatment option for women with irreversible ovarian function loss or primary ovarian failure [132] showing a pregnancy and implantation rates in oocyte donation cycles higher than in standard IVF/ICSI cycles [133]. In these cases, the support of the luteal is an essential prerequisite. In addition, as explained above, more and more frozen-thawed embryo transfers (FET) are employed with the aim of avoiding OHSS in high-risk patients. FET makes it possible for the embryos generated by IVF and ICSI to be stored and utilized later, with a segmentation of the IVF cycle, separating the ovarian stimulation and trigger from the fresh transfer, and vitrifying all embryos for subsequent transfer in frozen thawed cycles.

Moreover, not only for reducing the OHSS risk, in recent years the amount of FET cycles has dramatically increased due to the trend towards transferring fewer embryos after a fresh IVF cycle, and as a result of improved laboratory techniques [134, 135] until to the emerging “freeze-all” policy as an alternative to fresh embryo transfer (ET) to improve IVF outcomes [106]. A recent prospective, observational, cohort study has suggested that the “freeze-all” policy is advantageous when compared with fresh ETs [136]. Clinical (RR 1.29, 95 % CI 1.05 to 1.59) and ongoing pregnancy (RR 1.28, 95 % CI 1.01 to 1.62) rates were significantly better after FET [136]. In addition, a recent meta-analysis of observational studies has shown that singleton pregnancies after FET are associated with a lower risk of obstetric outcomes, such as small for gestational age baby (RR 0.45, 95 % CI 0.30 to 0.66) and preterm birth (RR 0.84, 95 % CI 0.78 to 0.90) [137].

Unlike the complex ovarian stimulation protocols of IVF and ICSI cycles, in the FET cycles, the primary target is limited to adequate preparation of the endometrium to receive the thawed embryos. The endometrial preparation can be achieved by means of two different methods, a natural cycle (NC) which consists of using endogenous sex steroids production from developing follicles in patients with regular ovulatory cycles and the artificial cycles (AC), where the endocrine preparation of the endometrium is achieved by sequential administration of estrogens and progestogens to mimic a normal cycle, more suitable for women without regular ovulatory cycles. The entire success of the NC-FET cycle depends on the correct identification of ovulation and calculation of the likely subsequent period of optimal endometrial receptivity [138, 139]. The identification of ovulation is based on detecting the spontaneous LH surge, therefore LH levels need to be regularly (daily) monitored in blood or urine samples, because it is assumed that ovulation will occur 36 – 40 h after LH peak [140]. Unfortunately, the LH surges in urine lag up to 21 h behind the appearance of the blood surge, creating difficulties in correct assessing of data. To overcome these problems, a modified-NC (mNC) has been proposed, which consists of hCG triggering when dominant follicle is of optimal size (>17 mm), after an ultrasound evaluation to ensure the appropriate timing of hCG administration.

In the AC-FET the timing of embryo thawing and transfer is planned according to the moment of progesterone supplementation but the sequential administration of exogenous sex steroids does not guarantee complete pituitary suppression (at least 5 % of early luteinizations, [141]), putting the endometrium at risk of early exposure to progesterone. Also for the AC cycle, a modified AC-FET (mAC) has been proposed consisting in the co-treatment with GnRH agonist in order to down-regulate the pituitary and prevent early follicular luteinizations [142].

A recent meta-analysis regarding the optimal endometrial preparation method in FET revealed no significant advantage of one specific approach to prepare the endometrium for FET in terms of clinical pregnancy rates or live birth rates [142]. In particular, no significant benefit of true NC-FET versus modified NC-FET with regard to clinical pregnancy (OR 0.91, 95 % CI 0.74 to 1.10), ongoing pregnancy (OR 1.0, 95 % CI 0.66 to 1.60) or live birth (OR 1.0, 95 % CI 0.63 to 1.60) was observed. Also luteal phase support was evaluated as potential influencing factor on pregnancy rate but the meta-analysis concludes that currently there is too little evidence supporting a positive effect of luteal phase support in patients undergoing NC-FET [132]. More recently, a retrospective study evaluating 228 patients showed that progesterone for luteal phase supplementation decreases miscarriage rate and improves live birth rate in NC-FET cycles [143] but, due to different study designs, the overall conflicting results of the available studies, do not make it possible to conclude a positive effect of luteal phase support in patients undergoing NC-FET [144].

Other issues of discussion concerned the different timing of progesterone starting and routes of administration in donor and/or FET cycles. A Cochrane review [145] analyzed the most effective endometrial preparation for women undergoing embryo transfer with frozen embryos and with embryos derived from donated oocytes. Starting progesterone on the day of oocyte aspiration (OR 1.92, 95 % CI 1.08 to 3.42) or on the day after (OR 1.81, 95 % CI 1.01 to 3.24) led to higher pregnancy rate than when progesterone was started the day before oocyte retrieval [145]. However, no difference was found between starting progesterone the day of oocyte aspiration or the day after (OR 0.94, 95 % CI 0.53 to 1.68) [95, 145]. Finally, no differences were found (OR 0.75, 95 % CI 0.24 to 2.34) between starting one or two days before the oocyte retrieval when the transfer is performed on blastocyst stage [145].

As regards progesterone route of administration, no statistical significant difference was detected in live-birth and clinical pregnancy rate between vaginal and intramuscular administration [145]. On the contrary, in a retrospective cohort study [146] women supplemented with vaginal progesterone gel had significantly lower rate of clinical pregnancy (36.9 % vs. 51.1 %, respectively) and live birth (24.4 % vs. 39.1 %, respectively) compared with intramuscular progesterone. The benefit of intramuscular route could be explained with the higher and continuous serum progesterone levels that act relaxing uterus and reducing the frequency of endometrial waves in the luteal phase that are associated with lower pregnancy rates [73]. Moreover, the booster injection of intramuscular progesterone (50 mg IM, once every 3 days) did not improved the pregnancy rates of patients who received vaginal progesterone (100 mg, three times daily) [147].

The worst results in vaginal progesterone group probably depend on the dosage of vaginal progesterone. In a retrospective study [148] patients treated with vaginal progesterone gel twice daily had a lower risk of pregnancy loss compared with women treated once a day (43.7 % vs. 67.4 %, respectively) resulting in a delivery rate (20.5 % vs. 8.7 %, respectively) more than two-fold higher [148]. However, considering recipients who received vitrified blastocysts on day 6, no statistical differences were seen in implantation and pregnancy rate between vaginal and intramuscular progesterone luteal phase support [149].

Very few data are available regarding the best dosage of progesterone administration. A recent study seems to demonstrate that the optimal dose of intramuscular progesterone to for luteal phase support ranges between 50 and 100 mg/day [150]. In fact, that dose lets to reach progesterone blood levels similar to those observed during the mid-luteal phase of a spontaneous ovulatory cycle. Moreover, an increase in progesterone dosage could be useful in donor and/or frozen-thawed AC cycles when the endometrial pattern at ultrasound is non-homogeneous hyperechoic in mid-luteal phase [151].

## Conclusions

Current data do not completely explain the clinical significance of LPD and its treatment. Several mechanisms of the implantation physiology and of “implantation window” are still not completely understood and are difficult to study owing to the lack of adequate tools for assessing endometrial receptivity. Future perspectives should concern endometrial competence evaluation, with the aim of revealing its physiologic mechanisms and to identify a correct diagnostic test for LPD in order to recognize it when it occurs in infertile couples.

In fact, LPD, is not univocally considered an infertility factor, and, even if it can be associated with infertility, it should be treated only in selected cases. The correct approach to the treatment of LPD is the identification and correction of any underlying condition, such as hypothalamic amenorrhea, thyroid and prolactin disorders, obesity and PCOS and during COS for IVF cycles. The available scientific literature is in agreement that in all gonadotropin COS/COH cycles for IVF and non-IVF cycles, progesterone supplementation is needed for luteal phase support, showing an improvement of the main reproductive outcomes. The protocols of luteal phase support with progesterone change according to the type of protocol of ovarian stimulation, i.e. conventional or non-conventional COH. Among different routes of administrations, there are no statistically significant differences between intramuscular progesterone and the others routes (vaginal, oral and rectal) for IVF cycles, fresh and FET, and the optimal timing and doses are not yet known.

Much of the current scientific evidence is based on reviews and meta-analyses of observational studies and on few RCTs, thus the future perspectives should take into consideration the implementation of randomized trials and the evaluation of the promising efficacy of subcutaneous progesterone at high-doses in the new COH cycles in which GnRH agonist are used to trigger multiple ovulation. Thus, at the moment, the scientific debate still remains open regarding progesterone administration protocols, in particular routes of administration, dose and timing and the potential association with other drugs, and further research is still needed.

## Abbreviations

ART: Assisted reproduction technology
LPD: Luteal phase deficiency
HCG: Human chorionic gonadotropin
NCS: Nucleolar channel system
PCOS: Polycystic ovarian syndrome
IVF: In-vitro fertilization
COS: Controlled ovarian stimulation
FGF: Fibroblast growth factors
FSH: Follicular stimulating hormone
LH: Luteinizing hormone
CC: Clomiphene citrate
AI: Aromatase inhibitor
IUI: Intrauterine insemination
OHSS: Ovarian hyperstimulation syndrome
COH: Controlled ovarian hyperstimulation
GnRH: Gonadotropin releasing hormone
ICSI: Intracytoplasmic sperm injection
FET: Frozen-thawed embryo transfer
NC: Natural cycle
AC: Artificial cycle

## References

[1] Practice Committee of the American Society for Reproductive Medicine (2015). Current clinical irrelevance of luteal phase deficiency: a committee opinion. Fertil Steril.

[2] Jones GE (1949). Some newer aspects of the management of infertility. J Am Med Assoc..

[3] Casper RF (2011). It’s time to pay attention to the endometrium. Fertil Steril.

[4] Cakmak H, Taylor HS (2011). Implantation failure: molecular mechanisms and clinical treatment. Hum Reprod Update.

[5] Simon C, Martin JC, Pellicer A (2000). Paracrine regulators of implantation. Baillieres Best Pract Res Clin Obstet Gynaecol.

[6] Strowitzki T, Germeyer A, Popovici R, von Wolff M (2006). The human endometrium as a fertility-determining factor. Hum Reprod Update.

[7] Wang H, Dey SK (2006). Roadmap to embryo implantation: clues from mouse models. Nat Rev Genet.

[8] Dey SK, Lim H, Das SK, Reese J, Paria BC, Daikoku T (2004). Molecular cues to implantation. Endocr Rev.

[9] Zapantis G, Szmyga MJ, Rybak EA, Meier UT (2013). Premature formation of nucleolar channel systems indicates advanced endometrial maturation following controlled ovarian hyperstimulation. Hum Reprod..

[10] Druckmann R, Druckmann MA (2005). Progesterone and the immunology of pregnancy. J Steroid Biochem Mol Biol.

[11] Szekeres-Bartho J, Wilczynski JR, Basta P, Kalinka J (2008). Role of progesterone and progestin therapy in threatened abortion and preterm labour. Front Biosci.

[12] Simoncini T, Caruso A, Garibaldi S, Fu XD, Giretti MS, Baldacci C (2006). Activation of nitric oxide synthesis in human endothelial cells using nomegestrol acetate. Obstet Gynecol.

[13] Hill MJ, Whitcomb BW, Lewis TD, Wu M, Terry N, DeCherney AH (2013). Progesterone luteal support after ovulation induction and intrauterine insemination: a systematic review and meta-analysis. Fertil Steril.

[14] Csapo AI, Pulkkinen MO, Wiest WG (1973). Effects of luteectomy and progesterone replacement therapy in early pregnant patients. Am J Obstet Gynecol.

[15] Filicori M, Flamigni C, Meriggiola MC, Ferrari P, Michelacci L, Campaniello E (1991). Endocrine response determines the clinical outcome of pulsatile gonadotropin-releasing hormone ovulation induction in different ovulatory disorders. J Clin Endocrinol Metab..

[16] Homer HA, Li TC, Cooke ID (2000). The septate uterus: a review of management and reproductive outcome. Fertil Steril..

[17] Afifi K, Anand S, Nallapeta S, Gelbaya TA (2010). Management of endometrial polyps in subfertile women: a systematic review. Eur J Obstet Gynecol Reprod Biol..

[18] Pritts EA, Parker WH, Olive DL (2009). Fibroids and infertility: an updated systematic review of the evidence. Fertil Steril..

[19] Johnson N, van Voorst S, Sowter MC, Strandell A, Mol BW. Surgical treatment for tubal disease in women due to undergo in vitro fertilisation. Cochrane Database Syst Rev. 2010; doi: 10.1002/14651858 10.1002/14651858.CD002125.pub3 PMC7154223 20091531

[20] Duffy JM, Arambage K, Correa FJ, Olive D, Farquhar C, Garry R, et al. Laparoscopic surgery for endometriosis. Cochrane Database Syst Rev. 2014; doi: 10.1002/14651858. 10.1002/14651858.CD011031.pub2 24696265

[21] Perez-Medina T, Bajo-Arenas J, Salazar F, Redondo T, Sanfrutos L, Alvarez P (2005). Endometrial polyps and their implication in the pregnancy rates of patients undergoing intrauterine insemination: a prospective, randomized study. Hum Reprod..

[22] Noyes RW, Haman JO (1953). Accuracy of endometrial dating; correlation of endometrial dating with basal body temperature and menses. Fertil Steril..

[23] Nikas G (1999). Pinopodes as markers of endometrial receptivity in clinical practice. Hum Reprod..

[24] Sharkey AM, Smith SK (2003). The endometrium as a cause of implantation failure. Best Pract Res Clin Obstet Gynaecol..

[25] Lessey BA, Killam AP, Metzger DA, Haney AF, Greene GL, McCarty KS (1988). Immunohistochemical analysis of human uterine estrogen and progesterone receptors throughout the menstrual cycle. J Clin Endocrinol Metab..

[26] Shoupe D, Mishell DR, Lacarra M, Lobo RA, Horenstein J, D’Ablaing G (1989). Correlation of endometrial maturation with four methods of estimating day of ovulation. Obstet Gynecol..

[27] Lindhard A, Ravn V, Bentin-Ley U, Horn T, Bangsboell S, Rex S (2006). Ultrasound characteristics and histological dating of the endometrium in a natural cycle in infertile women compared with fertile controls. Fertil Steril..

[28] Ng EH, Chan CC, Tang OS, Yeung WS, Ho PC (2009). Changes in endometrial and subendometrial blood flow in IVF. Reprod Biomed Online.

[29] Talbi S, Hamilton AE, Vo KC, Tulac S, Overgaard MT, Dosiou C (2006). Molecular phenotyping of human endometrium distinguishes menstrual cycle phases and underlying biological processes in normo-ovulatory women. Endocrinol.

[30] Jordan J, Craig K, Clifton DK, Soules MR (1994). Luteal phase defect: the sensitivity and specificity of diagnostic methods in common clinical use. Fertil Steril..

[31] Filicori M, Butler JP, Crowley WF (1984). Neuroendocrine regulation of the corpus luteum in the human. Evidence for pulsatile progesterone secretion. J Clin Invest.

[32] McGovern PG, Myers ER, Silva S, Coutifaris C, Carson SA, Legro RS (2004). Absence of secretory endometrium after false-positive home urine luteinizing hormone testing. Fertil Steril.

[33] Lessey BA (2011). Assessment of endometrial receptivity. Fertil Steril..

[34] Haouzi D, Assou S, Mahmoud K, Tondeur S, Rème T, Hedon B (2009). Gene expression profile of human endometrial receptivity: comparison between natural and stimulated cycles for the same patients. Hum Reprod..

[35] Tapia-Pizarro A, Figueroa P, Brito J, Marín JC, Munroe DJ, Croxatto HB (2014). Endometrial gene expression reveals compromised progesterone signaling in women refractory to embryo implantation. Reprod Biol Endocrinol..

[36] Anteby EY, Natanson-Yaron S, Hamani Y, Sciaki Y, Goldman-Wohl D, Greenfield C (2005). Fibroblast growth factor-10 and fibroblast growth factor receptors 1–4: expression and peptide localization in human decidua and placenta. Eur J Obstet Gynecol Reprod Biol..

[37] Sak ME, Gul T, Evsen MS, Soydinc HE, Sak S, Ozler A (2013). Fibroblast growth factor-1 expression in the endometrium of patients with repeated implantation failure after in vitro fertilization. Eur Rev Med Pharmacol Sci..

[38] National Collaborating Centre for Women's and Children's Health (UK) (2004). Fertility: Assessment and Treatment for People with Fertility Problems.

[39] Daly DC, Walters CA, Soto-Albors CE, Riddick DH (1983). Endometrial biopsy during treatment of luteal phase defects is predictive of therapeutic outcome. Fertil Steril..

[40] Tavaniotou A, Albano C, Smitz J, Devroey P (2002). Impact of ovarian stimulation on corpus luteum function and embryonic implantation. J Reprod Immunol..

[41] Haas DM, Ramsey PS (2013). Progestogen for preventing miscarriage. Cochrane Database Syst Rev..

[42] Wahabi HA, Fayed AA, Esmaeil SA, Al Zeidan RA (2011). Progestogen for treating threatened miscarriage. Cochrane Database Syst Rev..

[43] Palomba S (2015). Aromatase inhibitors for ovulation induction. J Clin Endocrinol Metab.

[44] Palomba S, Orio F, Falbo A, Russo T, Tolino A, Zullo F (2006). Effects of metformin and clomiphene citrate on ovarian vascularity in patients with polycystic ovary syndrome. Fertil Steril..

[45] Miralpeix E, González-Comadran M, Solà I, Manau D, Carreras R, Checa MA (2014). Efficacy of luteal phase support with vaginal progesterone in intrauterine insemination: a systematic review and meta-analysis. J Assist Reprod Genet..

[46] Gerli S, Gholami H, Manna C, Di Frega AS, Vitiello C, Unfer V (2000). Use of ethinyl estradiol to reverse the antiestrogenic effects of clomiphene citrate in patients undergoing intrauterine insemination: a comparative, randomized study. Fertil Steril..

[47] Elkind-Hirsch KE, Darensbourg C, Creasy G, Gipe D (2005). Conception rates in clomiphene citrate cycles with and without hormone supplementation: a pilot study. Curr Med Res Opin.

[48] Moini A, Ahmadi F, Jahangiri N, Ahmadi J, Akhoond MR. A randomized controlled trial evaluating the effect of ethinyl estradiol during clomiphene citrate cycles among women with polycystic ovary syndrome. Int J Gynaecol Obstet. 2015;131:129–32. 10.1016/j.ijgo.2015.06.032 26391671

[49] Elguero S, Wyman A, Hurd WW, Barker N, Patel B, Liu JH (2015). Does progesterone supplementation improve pregnancy rates in clomiphene citrate and intrauterine insemination treatment cycles?. Gynecol Endocrinol..

[50] Palomba S, Falbo A, Zullo F, Orio F (2009). Evidence-based and potential benefits of metformin in the polycystic ovary syndrome: a comprehensive review. Endocr Rev..

[51] Palomba S, Falbo A, Russo T, Orio F, Tolino A, Zullo F (2010). Systemic and local effects of metformin administration in patients with polycystic ovary syndrome (PCOS): relationship to the ovulatory response. Hum Reprod..

[52] Fleming R, Hopkinson ZE, Wallace AM, Greer IA, Sattar N (2002). Ovarian function and metabolic factors in women with oligomenorrhea treated with metformin in a randomized double blind placebo-controlled trial. J Clin Endocrinol Metab..

[53] Legro RS, Brzyski RG, Diamond MP, Coutifaris C, Schlaff WD, Alvero R (2014). Letrozole versus clomiphene for in- fertility in the polycystic ovary syndrome. N Engl J Med.

[54] Franik S, Kremer JA, Nelen WL, Farquhar C, Marjoribanks J (2015). Aromatase inhibitors for subfertile women with polycystic ovary syndrome: summary of a Cochrane review. Fertil Steril..

[55] Biberoglu EH, Tanrikulu F, Erdem M, Erdem A, Biberoglu KO (2015). Luteal phase support in intrauterine insemination cycles: a prospective randomized study of 300 mg versus 600 mg intravaginal progesterone tablet. Gynecol Endocrinol..

[56] Munne S, Magli C, Adler A, Wright G, de Boer K, Mortimer D (1997). Treatment-related chromosome abnormalities in human embryos. Hum Reprod..

[57] Katz-Jaffe MG, Trounson AO, Cram DS (2005). Chromosome 21 mosaic human preimplantation embryos predominantly arise from diploid conceptions. Fertil Steril..

[58] Baart EB, Martini E, Eijkemans MJ, Van Opstal D, Beckers NG, Verhoeff A (2007). Milder ovarian stimulation for in-vitro fertilization reduces aneuploidy in the human preimplantation embryo: a randomized controlled trial. Hum Reprod..

[59] Verberg MF, Macklon NS, Nargund G, Frydman R, Devroey P, Broekmans FJ (2009). Mild ovarian stimulation for IVF. Hum Reprod Update..

[60] Verberg MF, Eijkemans MJ, Macklon NS, Heijnen EM, Baart EB, Hohmann FP (2009). The clinical significance of the retrieval of a low number of oocytes following mild ovarian stimulation for IVF: a meta-analysis. Hum Reprod Update..

[61] Devroey P, Bourgain C, Macklon NS, Fauser BC (2004). Reproductive biology and IVF: ovarian stimulation and endometrial receptivity. Trends Endocrinol Metab..

[62] Fauser BC, Devroey P (2003). Reproductive biology and IVF: ovarian stimulation and luteal phase consequences. Trends Endocrinol Metab..

[63] Casper RF, Yen SS (1979). Induction of luteolysis in the human with a long-acting analog of luteinizing hormone-releasing factor. Science..

[64] Mais V, Kazer RR, Cetel NS, Rivier J, Vale W, Yen SS (1986). The dependency of folliculogenesis and corpus luteum function on pulsatile gonadotropin secretion in cycling women using a gonadotropin-releasing hormone antagonist as a probe. J Clin Endocrinol Metab..

[65] Beckers NG, Macklon NS, Eijkemans MJ, Ludwig M, Felberbaum RE, Diedrich K (2003). Nonsupplemented luteal phase characteristics after the administration of recombinant human chorionic gonadotropin, recombinant luteinizing hormone, or gonadotropin-releasing hormone (GnRH) agonist to induce final oocyte maturation in in vitro fertilization patients after ovarian stimulation with recombinant follicle-stimulating hormone and GnRH antagonist cotreatment. J Clin Endocrinol Metab..

[66] Beckers NG, Laven JS, Eijkemans MJ, Fauser BC (2000). Follicular and luteal phase characteristics following early cessation of gonadotrophin-releasing hormone agonist during ovarian stimulation for in-vitro fertilization. Hum Reprod..

[67] Andersen YC, Andersen VK (2014). Improving the luteal phase after ovarian stimulation: reviewing new options. Reprod Biomed Online.

[68] National Collaborating Centre for Women's and Children's Health (UK) (2013). Fertility: Assessment and Treatment for People with Fertility Problems.

[69] Practice Committee of Society for Assisted Reproductive Technology; Practice Committee of American Society for Reproductive Medicine (2008). Guidelines on number of embryos transferred. Fertil Steril.

[70] Van der Linden M, Buckingham K, Farquhar C, Kremer JAM, Metwally M (2015). Luteal phase support for assisted reproduction cycles. Cochrane Database Syst Rev..

[71] Van der Linden M, Buckingham K, Farquhar C, Kremer JAM, Metwally M (2011). Luteal phase support for assisted reproduction cycles. Cochrane Database Syst Rev..

[72] Cicinelli E, de Ziegler D, Bulletti C, Matteo MG, Schonauer LM, Galantino P (2000). Direct transport of progesterone from vagina to uterus. Obstet Gynecol..

[73] Casper RF (2014). Luteal phase support for frozen embryo transfer cycles: intramuscular or vaginal progesterone?. Fertil Steril..

[74] Tavaniotou A, Smitz J, Bourgain C, Devroey P (2000). Comparison between different routes of progesterone administration as luteal phase support in infertility treatments. Hum Reprod Update..

[75] Zarutskie PW, Phillips JA (2009). A meta-analysis of the route of administration of luteal phase support in assisted reproductive technology: vaginal versus intramuscular progesterone. Fertil Steril..

[76] Bourgain C, Devroey P, Van Waesberghe L, Smitz J, Van Steirteghem AC (1990). Effects of natural progesterone on the morphology of the endometrium in patients with primary ovarian failure. Hum Reprod..

[77] Smitz J, Devroey P, Faguer B, Bourgain C, Camus M, Van Steirteghem AC (1992). A prospective randomized comparison of intramuscular or intravaginal natural progesterone as a luteal phase and early pregnancy supplement. Hum Reprod..

[78] Yanushpolsky EH (2015). Luteal phase support in in vitro fertilization. Semin Reprod Med..

[79] Gizzo S, Andrisani A, Esposito F, Noventa M, Di Gangi S, Angioni S (2014). Which luteal phase support is better for each IVF stimulation protocol to achieve the highest pregnancy rate? A superiority randomized clinical trial. Gynecol Endocrinol..

[80] Dal Prato L, Bianchi L, Cattoli M, Tarozzi N, Flamigni C, Borini A (2008). Vaginal gel versus intramuscular progesterone for luteal phase supplementation: a prospective randomized trial. Reprod Biomed Online..

[81] Doody KJ, Schnell VL, Foulk RA, Miller CE, Kolb BA, Blake EJ (2009). Endometrin for luteal phase support in a randomized, controlled, open-label, prospective in-vitro fertilization trial using a combination of Menopur and Bravelle for controlled ovarian hyperstimulation. Fertil Steril..

[82] Stadtmauer L, Silverberg KM, Ginsburg ES, Weiss H, Howard B (2013). Progesterone vaginal ring versus vaginal gel for luteal support with in vitro fertilization: a randomized comparative study. Fertil Steril..

[83] Lockwood G, Griesinger G, Cometti B, 13 European Centers (2014). Subcutaneous progesterone versus vaginal progesterone gel for luteal phase support in in vitro fertilization: a noninferiority randomized controlled study. Fertil Steril.

[84] Baker VL, Jones CA, Doody K, Foulk R, Yee B, Adamson GD (2014). A randomized, controlled trial comparing the efficacy and safety of aqueous subcutaneous progesterone with vaginal progesterone for luteal phase support of in vitro fertilization. Hum Reprod..

[85] De Ziegler D (2013). A randomized trial comparing the endometrial effects of daily subcutaneous administration of 25 mg and 50 mg of progesterone in aqueous preparation. Fertil Steril..

[86] Sator M, Radicioni M, Cometti B, Loprete L, Leuratti C, Schmidl D (2013). Pharmacokinetics and safety profile of a novel progesterone aqueous formulation administered by the s.c. route. Gynecol Endocrinol.

[87] Tesarik J, Hazout A, Mendoza-Tesarik R, Mendoza N, Mendoza C (2006). Beneficial effect of luteal-phase GnRH agonist administration on embryo implantation after ICSI in both GnRH agonist- and antagonist-treated ovarian stimulation cycles. Hum Reprod..

[88] Pirard C, Donnez J, Loumaye E (2005). GnRH agonist as novel luteal support: results of a randomized, parallel group, feasibility study using intranasal administration of buserelin. Hum Reprod..

[89] Pirard C, Donnez J, Loumaye E (2006). GnRH agonist as luteal phase support in assisted reproduction technique cycles: results of a pilot study. Hum Reprod..

[90] Connell MT, Szatkowski JM, Terry N, DeCherney AH, Props MT, Hill MK (2015). Timing luteal support in assisted reproductive technology: a systematic review. Fertil Steril..

[91] Mochtar MH, van Wely M, van der Veen F (2006). Timing luteal phase support in GnRH agonist down-regulated IVF/embryo transfer cycles. Hum Reprod..

[92] Sohn SH, Penzias AS, Emmi AM, Dubey AK, Layman LC, Reindollar RH (1999). Administration of progesterone before oocyte retrieval negatively affects the implantation rate. Fertil Steril..

[93] Williams SC, Oehninger S, Gibbons WE, van Cleave WC, Muasher SJ (2001). Delaying the initiation of progesterone supplementation results in decreased pregnancy rates after in vitro fertilization: a randomized, prospective study. Fertil Steril..

[94] Fanchin R, Righini C, de Ziegler D, Olivennes F, Ledee N, Frydman R (2001). Effects of vaginal progesterone administration on uterine contractility at the time of embryo transfer. Fertil Steril..

[95] Escribá MJ, Bellver J, Bosch E, Sánchez M, Pellicer A, Remohí J (2006). Delaying the initiation of progesterone supplementation until the day of fertilization does not compromise cycle outcome in patients receiving donated oocytes: a randomized study. Fertil Steril..

[96] Liu XR, Mu HQ, Shi Q, Xiao XQ, Qi HB (2012). The optimal duration of progesterone supplementation in pregnant women after IVF/ICSI: a meta-analysis. Reprod Biol Endocrinol..

[97] Devroey P, Adriaensen P (2011). OHSS Free Clinic. Facts Views Vision..

[98] Iliodromiti S, Blockeel C, Tremellen KP, Fleming R, Tournaye H, Humaidan P (2013). Consistent high clinical pregnancy rates and low ovarian hyperstimulation syndrome rates in high-risk patients after GnRH agonist triggering and modified luteal support: a retrospective multicentre study. Hum Reprod..

[99] Corbett S, Shmorgun D, Claman P (2014). Reproductive Endocrinology Infertility Committee, Healey S, Gysler M. The prevention of ovarian hyperstimulation syndrome. J Obstet Gynaecol Can.

[100] Shapiro BS, Daneshmand ST, Garner FC, Aguirre M, Hudson C, Thomas S (2011). Evidence of impaired endometrial receptivity after ovarian stimulation for in vitro fertilization: a prospective randomized trial comparing fresh and frozen-thawed embryo transfer in normal responders. Fertil Steril..

[101] Shapiro BS, Daneshmand ST, Garner FC, Aguirre M, Hudson C, Thomas S (2011). Evidence of impaired endometrial receptivity after ovarian stimulation for in vitro fertilization: a prospective randomized trial comparing fresh and frozen-thawed embryo transfer in high responders. Fertil Steril..

[102] Roque M, Lattes K, Serra S, Sola I, Geber S, Carreras R (2013). Fresh embryo transfer versus frozen embryo transfer in in vitro fertilization cycles: a systematic review and meta-analysis. Fertil Steril..

[103] Roque M (2015). Freeze-all policy: is it time for that?. J Assist Reprod Genet..

[104] Devroey P, Polyzos NP, Blockeel C (2011). An OHSS-free clinic by segmentation of IVF treatment. Hum Reprod..

[105] Pinborg A, Henningsen AA, Loft A, Malchau SS, Forman J, Andersen AN (2014). Large baby syndrome in singletons born after frozen embryo transfer (FET): is it due to maternal factors or the cryotechnique?. Hum Reprod..

[106] Maheshwari A, Bhattacharya S (2013). Elective frozen replacement cycles for all: ready for prime time?. Hum Reprod..

[107] Segal S, Casper RF (1992). Gonadotropin-releasing hormone agonist versus human chorionic gonadotropin for triggering follicular maturation in in vitro fertilization. Fertil Steril..

[108] Humaidan P, Engmann L, Benadiva C (2015). Luteal phase supplementation after gonadotropin-releasing hormone agonist trigger in fresh embryo transfer: the American versus European approaches. Fertil Steril..

[109] Humaidan P, Bredkjaer HE, Bungum L (2005). GnRH agonist (buserelin) or hCG for ovulation induction in GnRH antagonist IVF/ICSI cycles: a prospective randomised study. Hum Reprod..

[110] Humaidan P, Papanikolaou EG, Tarlatzis BC (2009). GnRHa to trigger final oocyte maturation: a time to reconsider. Hum Reprod..

[111] Gonen Y, Balakier H, Powell W, Casper RF (1990). Use of gonadotropin-releasing hormone agonist to trigger follicular maturation for in vitro fertilization. J Clin Endocrinol Metab..

[112] Itskovitz J, Boldes R, Levron J, Erlik Y, Kahana L, Brandes JM (1991). Induction of preovulatory luteinizing hormone surge and prevention of ovarian hyperstimulation syndrome by gonadotropin-releasing hormone agonist. Fertil Steril..

[113] Humaidan P, Kol S, Papanikolaou EG, Copenhagen GnRH Agonist Triggering Workshop Group (2011). GnRH agonist for triggering of final oocyte maturation: time for a change of practice?. Hum Reprod Update.

[114] Hull MG, Savage PE, Bromham DR, Ismail AA, Morris AF (1982). The value of a single serum progesterone measurement in the midluteal phase as a criterion of a potentially fertile cycle (‘ovulation’) derived from treated and untreated conception cycles. Fertil Steril.

[115] Yen SS, Llerena O, Little B, Pearson OH (1968). Disappearance rates of endogenous luteinizing hormone and chorionic gonadotropin in man. J Clin Endocrinol Metab..

[116] Acevedo B, Gomez-Palomares JL, Ricciarelli E, Hernández ER (2006). Triggering ovulation with gonadotropin-releasing hormone agonists does not compromise embryo implantation rates. Fertil Steril..

[117] Borgbo T, Povlsen BB, Andersen CY, Borup R, Humaidan P, Grondahl ML (2013). Comparison of gene expression profiles in granulosa and cumulus cells after ovulation induction with either human chorionic gonadotropin or a gonadotropin-releasing hormone agonist trigger. Fertil Steril..

[118] Bermejo A, Cerrillo M, Ruiz-Alonso M, Blesa D, Simòn C, Pellicer A (2014). Impact of final oocyte maturation using gonadotropin-releasing hormone agonist triggering and different luteal support protocols on endometrial gene expression. Fertil Steril..

[119] Youssef MA, Van der Veen F, Al-Inany HG, Mochtar MH, Griesinger G, Nagi Mohesen M (2014). Gonadotropin-releasing hormone agonist versus HCG for oocyte triggering in antagonist-assisted reproductive technology. Cochrane Database Syst Rev..

[120] Youssef MA, Van der Veen F, Al-Inany HG, Griesinger G, Mochtar MH, Aboulfoutouh I (2011). Gonadotropin-releasing hormone agonist versus HCG for oocyte triggering in antagonist assisted reproductive technology cycles. Cochrane Database Syst Rev..

[121] Kol S, Humaidan P, Alsbjerg B, Engmann L, Benadiva L, Garcia-Velasco JA (2015). The updated Cochrane review 2014 on GnRH agonist trigger: repeating the same errors. Reprod Biomed Online..

[122] Shapiro BS, Daneshmand ST, Garner FC, Aguirr M, Thomas S (2008). Gonadotropin-releasing hormone agonist combined with a reduced dose of human chorionic gonadotropin for final oocyte maturation in fresh autologous cycles of in vitro fertilization. Fertil Steril..

[123] Shapiro BS, Daneshmand ST, Garner FC, Aguirre M, Hudson C (2011). Comparison of "triggers" using leuprolide acetate alone or in combination with low-dose human chorionic gonadotropin. Fertil Steril..

[124] Humaidan P, Polyzos NP, Alsbjerg B, Erb K, Mikkelsen AL, Elbaek HO (2013). GnRHa trigger and individualized luteal phase hCG support according to ovarian response to stimulation: two prospective randomized controlled multi-centre studies in IVF patients. Hum Reprod..

[125] Beck-Fruchter R, Weiss A, Lavee M, Geslevich Y, Shalev E (2012). Empty follicle syndrome: successful treatment in a recurrent case and review of the literature. Hum Reprod..

[126] Wang W, Zhang XH, Wang WH, Liu YL, Zhao LH, Xue SL (2011). The time interval between hCG priming and oocyte retrieval in ART program: a meta-analysis. J Assist Reprod Genet..

[127] Orvieto R, Gan R. Triggering final follicular maturation- hCG, GnRH-agonist or both, when and to whom? J Ovarian Res. 2015; in press. 10.1186/s13048-015-0187-6 PMC4546254 26293447

[128] Haas J, Zilberberg E, Dar S, Kedem A, Machtinger R, Orvieto R (2014). Co-administration of GnRH-agonist and hCG for final oocyte maturation (double trigger) in patients with low number of oocytes retrieved per number of preovulatory follicles-a preliminary report. J Ovarian Res..

[129] Zilberberg E, Haas J, Dar S, Kedem A, Machtinger R, Orvieto R (2015). Co-administration of GnRH-agonist and hCG for final oocyte maturation in patients with low proportion of mature oocytes. Gynecol Endocrinol..

[130] Humaidan P, Polyzos NP (2014). Human chorionic gonadotropin vs. gonadotropin-releasing hormone agonist trigger in assisted reproductive technology--"the king is dead, long live the king!". Fertil Steril.

[131] Humaidan P, Alsbjerg B (2014). GnRHa trigger for final oocyte maturation: is HCG trigger history?. Reprod Biomed Online..

[132] Liu K, Case A, Reproductive Endocrinology and Infertility Committee, Family Physicians Advisory Committee, Maternal-Fetal Medicine Committee, Executive and Council of the Society of Obstetricians (2011). Advanced reproductive age and fertility. J Obstet Gynaecol Can.

[133] Society for Assisted Reproductive Technology, American Society for Reproductive Medicine (2004). Assisted reproductive technology in the United States: 2000 results generated from the American Society for Repro- ductive Medicine/Society for Assisted Reproductive Technology Registry. Fertil Steril..

[134] JOINT SOGC-CFAS (2008). Guidelines for the number of embryos to transfer following in vitro fertilization No. 182, September 2006. Int J Gynaecol Obstet.

[135] Min JK, Hughes E, Young D, Gysler M, Hemmings R, Cheung AP (2010). Elective single embryo transfer following in vitro fertilization. J Obstet Gynaecol Can..

[136] Roque M, Valle M, Guimaràes F, Sampaio M, Geber S (2015). Freeze-all policy: fresh vs. frozen-thawed embryo transfer. Fertil Steril.

[137] Maheshwari A, Pandey S, Shetty A, Hamilton M, Bhattacharya S (2012). Obstetric and perinatal outcomes in singleton pregnancies resulting from the transfer of frozen thawed versus fresh embryos generated through in vitro fertilization treatment: a systematic review and meta-analysis. Fertil Steril..

[138] Harper MJ (1992). The implantation window. Baillieres Clin Obstet Gynaecol..

[139] Tabibzadeh S (1998). Molecular control of the implantation window. Hum Reprod Update..

[140] Andersen AG, Als-Nielsen B, Hornnes PJ, Franch Andersen L (1995). Time interval from human chorionic gonadotrophin (HCG) injection to follicular rupture. Hum Reprod..

[141] El-Toukhy T, Taylor A, Khalaf Y, Al-Darazi K, Rowell P, Seed P (2004). Pituitary suppression in ultrasound-monitored frozen embryo replacement cycles. A randomised study. Hum Reprod.

[142] Groenewoud ER, Cantineau AE, Kollen BJ, Macklon NS, Cohlen BJ (2013). What is the optimal means of preparing the endometrium in frozen-thawed embryo transfer cycles? A systematic review and meta-analysis. Hum Reprod Update..

[143] Kim CH, Lee YJ, Lee KH, Kwon SK, Kim SH, Chae HD (2014). The effect of luteal phase progesterone supplementation on natural frozen-thawed embryo transfer cycles. Obstet Gynecol Sci..

[144] Ortega I, Velasco JA (2015). Progesterone supplementation in the frozen embryo transfer cycle. Curr Opin Obstet Gynecol..

[145] Glujovsky D, Pesce R, Fiszbajn G, Sueldo C, Hart RJ, Ciapponi A (2010). Endometrial preparation for women undergoing embryo transfer with frozen embryos or embryos derived from donor oocytes. Cochrane Database Syst Rev..

[146] Kaser DJ, Ginsburg ES, Missmer SA, Correia KF, Racowsky C (2012). Intramuscular progesterone versus 8 % Crinone vaginal gel for luteal phase support for day 3 cryopreserved embryo transfer. Fertil Steril..

[147] Feinberg EC, Beltsos AN, Nicolaou E, Marut EL, Uhler ML (2013). Endometrin as luteal phase support in assisted reproduction. Fertil Steril..

[148] Alsbjerg B, Polyzos NP, Elbaek HO, Povlsen BB, Andersen CY, Humaidan P (2013). Increasing vaginal progesterone gel supplementation after frozen-thawed embryo transfer significantly increases the delivery rate. Reprod Biomed Online..

[149] Shapiro DB, Pappadakis JA, Ellsworth NM, Hait HI, Nagy ZP (2014). Progesterone replacement with vaginal gel versus i.m. injection: cycle and pregnancy outcomes in IVF patients receiving vitrified blastocysts. Hum Reprod.

[150] Brady PC, Kaser DJ, Ginsburg ES, Ashby RK, Missmer SA, Correia KF (2014). Serum progesterone concentration on day of embryo transfer in donor oocyte cycles. J Assist Reprod Genet..

[151] Check JH, Dietterich C, Cohen R, Choe JK, Amui J, Brasile D (2010). Increasing the dosage of progesterone (P) supplemention from the mid-luteal phase in women not attaining a mid-luteal homogeneous hyperechogenic (HH) pattern with sonography improves pregnancy rates (PRS) following frozen embryo transfer (ET). Clin Exp Obstet Gynecol..

